# High-Flow Nasal Cannula Therapy in a Patient with Reperfusion Pulmonary Edema following Percutaneous Transluminal Pulmonary Angioplasty

**DOI:** 10.1155/2014/837612

**Published:** 2014-07-15

**Authors:** Kiyoshi Moriyama, Toru Satoh, Akira Motoyasu, Tomoki Kohyama, Mariko Kotani, Riichiro Kanai, Tadao Ando, Tomoko Yorozu

**Affiliations:** ^1^Department of Anesthesiology, Kyorin University School of Medicine, 6-20-2 Shinkawa, Mitaka, Tokyo 181-8611, Japan; ^2^Second Department of Internal Medicine, Kyorin University School of Medicine, 6-20-2 Shinkawa, Mitaka, Tokyo 181-8611, Japan

## Abstract

A 62-year-old woman with Wolff-Parkinson-White syndrome was with recent worsening of dyspnea to New York Heart Association functional status Class III. The patient was diagnosed as having central type chronic thromboembolic pulmonary hypertension. By cardiac catheterization, her mean pulmonary artery pressure was 53 mmHg with total pulmonary resistance 2238 dynes·sec·cm^−5^. After medical therapies with tadalafil, furosemide, ambrisentan, beraprost, and warfarin were initiated, percutaneous transluminal pulmonary angioplasty (PTPA) was performed. Following PTPA, life-threating hypoxemia resulting from postoperative reperfusion pulmonary edema developed. High-flow nasal cannula therapy (HFNC) was applied, and 100% oxygen at 50 L/min of flow was required to keep oxygenation. HFNC was continued for 3 days, and the patient was discharged on 8th postoperative day with SpO_2_ of 97% on 3 L/min of oxygen inhalation. Because of the simplicity of the technique, the lower cost of equipment, and remarkable patient tolerance to the treatment, we speculate that HFNC can take over the post of noninvasive ventilation as first-line therapy for patients with acute respiratory failure.

## 1. Introduction

For patients with chronic thromboembolic pulmonary hypertension (CTEPH), percutaneous transluminal pulmonary angioplasty (PTPA) was originally reported in 2001 in USA [1] and now developing in Japan [2, 3]. A troublesome complication associated with PTPA is reperfusion pulmonary edema, which almost always occurs within 48 h after vessel dilation [4]. We experienced a patient suffering from postoperative reperfusion pulmonary edema that was successfully managed with high-flow nasal cannula therapy (HFNC).

## 2. Case Presentation

A woman with Wolff-Parkinson-White syndrome noticed exercise intolerance and dyspnea at 61 years old. Her symptoms developed from New York Heart Association (NYHA) functional status Class II to III in 3 months. She was diagnosed to have CTEPH by lung perfusion scintigraphy and contrast CT image. Cardiac catheterization was performed and her mean pulmonary arterial pressure (mPAP) was 53 mmHg with total pulmonary resistance 2238 dynes·sec·cm^−5^. Her brain natriuretic peptide (BNP) level was 306.5 pg/dL, and she started to take tadalafil, furosemide, ambrisentan, beraprost, and warfarin. Because her symptoms worsened on supine position, ambulatory oxygen inhalation therapy while sleeping was started, and PTPA was scheduled.

**Figure 1:**
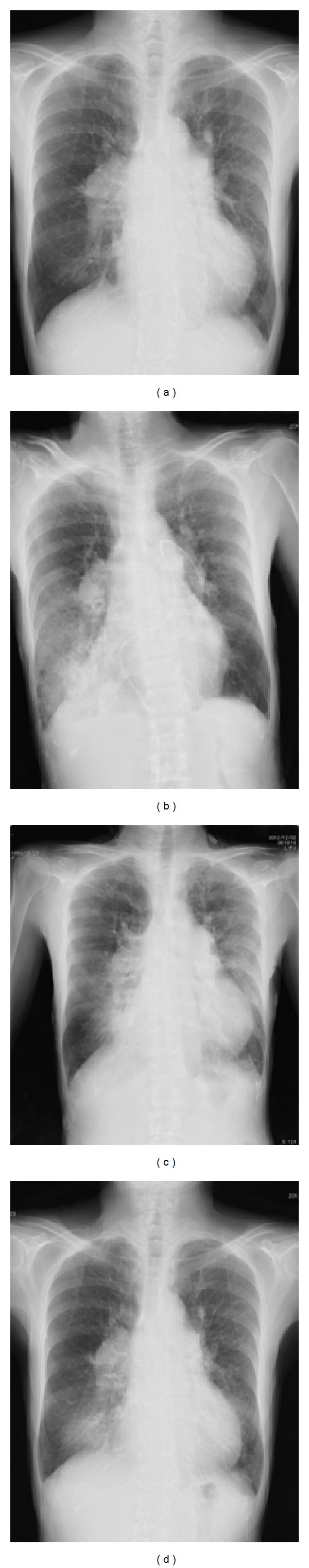
Chest X-ray obtained before percutaneous transluminal pulmonary angioplasty (a), on the first postoperative day (POD1) (b), POD4 (c), and POD8 (d). The patient had localized consolidation and atelectasis on her right lower lobe on POD1, 4, and 8, designating postsurgical pulmonary edema in the dilated segment.

On admission to our hospital, the patient's NYHA functional status was Class III. Preoperative cardiac catheterization showed that medical therapies decreased her mPAP from 53 to 42 mmHg, total pulmonary resistance from 2238 to 1223 dynes·sec·cm^−5^, and BNP level from 306.5 to 48.0 pg/dL. Her SaO_2_ was 93.7% and cardiothoracic ratio on chest X-ray was 56% (Figure 1(a)).

**Figure 2:**
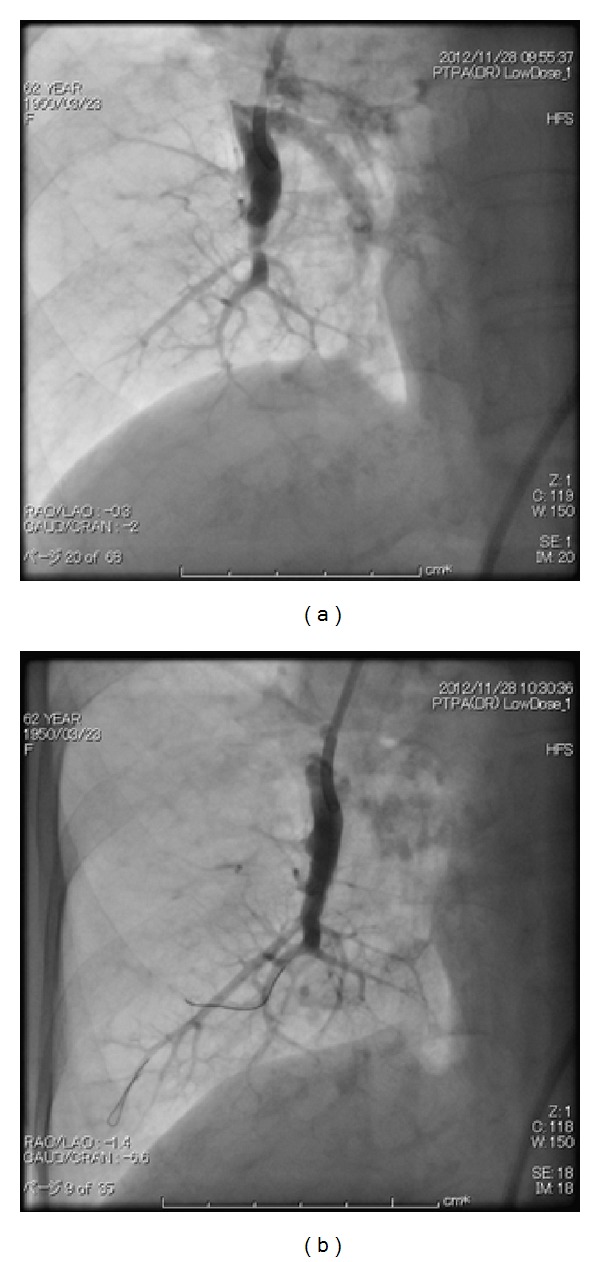
Initial percutaneous transluminal pulmonary angioplasty was performed for the right pulmonary artery (a). The A8 region of her right pulmonary artery was dilated by the balloon (plain old balloon atherectomy, b).

Initial PTPA was performed for her right pulmonary artery. The A8 region of her right pulmonary artery was dilated by the balloon (Figure 2). On admission to the ICU (day 0), her mPAP was 37 mmHg and her SpO_2_ was 99% with 3 L/min of oxygen inhalation through a nasal cannula with no complaint of dyspnea. Twelve hours after admission to the ICU, her SpO_2_ decreased to 77% with 5 L/min of oxygen inhalation, and pink frothy sputum was noticed with increases in mPAP up to 49 mmHg. To avoid hypoxemia and increases in PAP, HFNC was applied simultaneously with the administration of methylprednisolone (1000 mg/day) and furosemide (10 mg/day).

**Figure 3:**
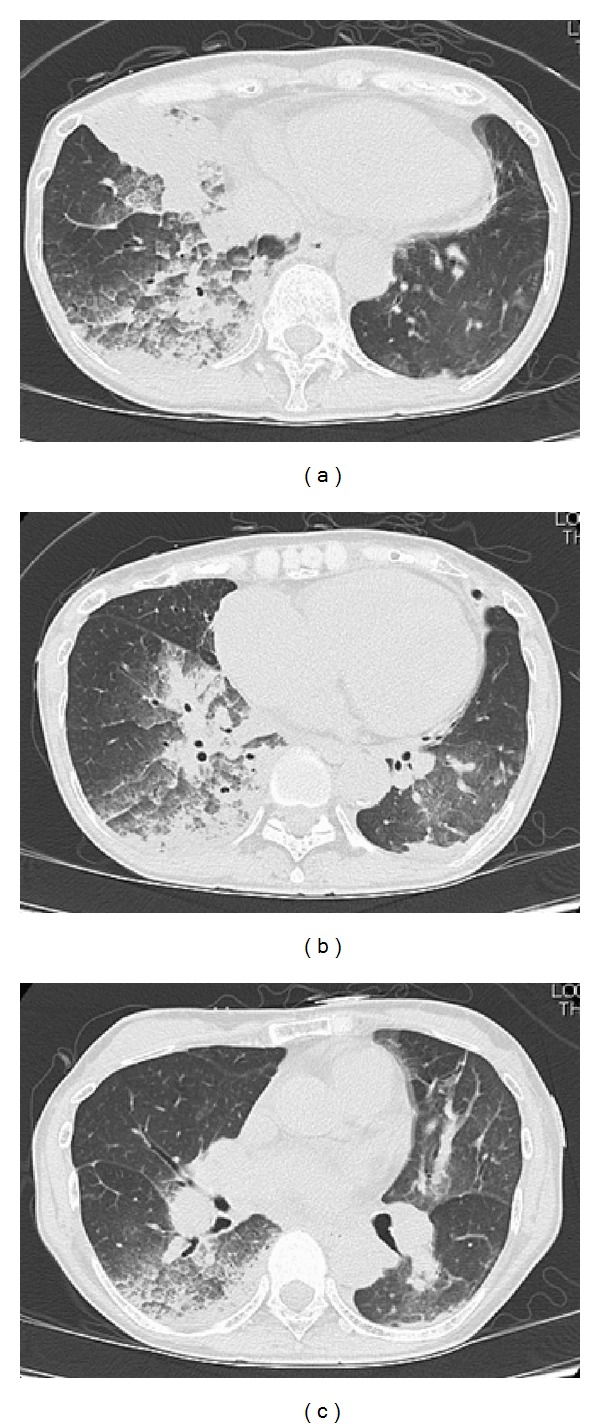
CT scan images obtained on the third postoperative day (POD3). The patient had localized consolidation on her right lower lobe with atelectasis, designating postsurgical pulmonary edema in the dilated segment.

The initial setting of HFNC was 90% oxygen at 35 L/min of flow. Immediately after applying HFNC, her SpO_2_ rose up to 93% and her mPAP decreased to 30 mmHg. On day 1, her chest X-ray revealed localized consolidation on her right lower lobe with atelectasis (Figure 1(b)). To avoid the development of pulmonary edema, the flow of HFNC was arisen up to 50 L/min.  Figure 3 shows CT scan images obtained on day 3. On day 3, with 50 L/min of flow unchanged, oxygen concentration was decreased to 50%. HFNC was finally discontinued on day 4 with her mPAP 34 mmHg (Figure 1(c)). The patient was discharged on the 8th postoperative day with SpO_2_ of 97% on 3 L/min of oxygen inhalation (Figure 1(d)).

## 3. Discussion

PTPA is a catheterization-based interventional management strategy for patients compromised with CTEPH, who are deemed nonsurgical or high-risk surgical candidates of pulmonary thromboendarterectomy [5]. Sugimura et al. reported that PTPA combined with conventional vasodilator treatment was effective in improving pulmonary hemodynamics in patients with distal-type CTEPH [6]. Feinstein et al. reported [1] that the reperfusion pulmonary edema was the most life-threatening postoperative complication following PTPA, and 3/18 patients required mechanical ventilation and 1 patient died 1 week after PTPA.

Typical management of reperfusion pulmonary edema includes diuretics and oxygen. In cases with worsening hypoxemia, noninvasive ventilation has been a first-line intervention to avoid endotracheal intubation [7]. Noninvasive ventilation is a technique to augment alveolar ventilation delivered by face mask, without endotracheal intubation. We previously reported a patient with postsurgical reperfusion pulmonary edema following PTPA [8]. This patient accepted a long duration of 16 days of noninvasive ventilation. However, because of mask tolerance, a long duration of noninvasive ventilation with an almost full-day dependence on ventilatory support is not applicable to all patients, even with slight sedative administration. Díaz-Lobato et al. reported a patient with acute respiratory failure of neuromuscular origin, who did not tolerate noninvasive ventilation but was treated successfully with HFNC [9].

HFNC oxygen therapy is a new alternative to conventional oxygen therapy [10]. HFNC delivers consistent and accurate oxygen concentrations and generates flows up to 60 L/min with optimal heat and humidity (37°C and 44 mg H_2_O/L) through a nasal cannula. The therapeutic advantages of HFNC are (1) preventing air dilution, (2) minimizing CO_2_ rebreathing, (3) generating moderate positive airway pressure [11], (4) increasing end-expiratory lung volumes and tidal volumes, and (5) maintaining the function of the mucociliary transport system, as well as (6) the simplicity of the technique, the lower cost of equipment, and remarkable patient tolerance to the treatment compared with endotracheal intubation or noninvasive ventilation [9]. These advantages benefited critical care patients with acute respiratory failure [12].

In this case, (1) preventing air dilution was necessary as the patient was severely hypoxemic. (2) Although hypercapnia was absent, decreasing PaCO_2_ was preferable to decrease mPAP, (3) generating moderate positive airway pressure was helpful to reduce pink frothy sputum due to reperfusion pulmonary edema. (4) Increasing end-expiratory lung volumes and tidal volumes was also helpful because the patient was also compromised with atelectasis. The patient was able to take medications and meals without hypoxemia and had no complaint of HFNC during 4 days.

Because of the simplicity of the technique, the lower cost of equipment, and remarkable patient tolerance to the treatment, we speculate that HFNC can take over the post of noninvasive ventilation as first-line therapy for patients with acute respiratory failure. Parke et al. compared HFNC with conventional high-flow face mask (HFFM) oxygen therapy [13] in 60 patients with hypoxic respiratory failure. They showed that HFNC significantly reduced desaturations and the rate of noninvasive ventilation. In their study, 10% of patients on HFNC required noninvasive ventilation due to worsening respiratory failure. When applying HFNC to patients with respiratory failure, we always need to consider noninvasive ventilation or endotracheal intubation in cases when increased dyspnea, respiratory fatigue, worsening gas exchange, or intolerance of allocated therapy continues.

In summary, we experienced a patient with postsurgical, reperfusion pulmonary edema following PTPA. Severe hypoxemia was successfully treated with HFNC. We speculate that HFNC can take over the post of noninvasive ventilation as first-line therapy for patients with acute respiratory failure.
